# Supplementary material for: Biology of the Extracellular Proteasome
Source: Biomolecules. 2022 Apr 21;12(5):619. doi: 10.3390/biom12050619 (PMC9139032; doi:10.3390/biom12050619)
Supplement: Supplementary file 1 [file biomolecules-12-00619-s001.zip › biomolecules-1680262-File S1.pdf]

**References of proteomic studies***

1. Amniotic fluid[1-9]
2. Ascites[10-12]
3. Bile[13-15]
4. Blood[16-23]
5. Bronchoalveolar Lavage[24-27]
6. Cancer interstitial fluids[11,28-30]
7. Cerebrospinal fluid[31-37]
8. Cerumen[38]
9. Cervicovaginal fluid[39-41]
10. Exhaled breath condensate[42-44]
11. Lung cancer pleural effusions[45-47]
12. Lymphatic drainage[23]
13. Lymph fluid[48-50]
14. Milk[51-56]
15. Nipple aspired fluid[57-59]
16. Pancreatic cyst fluid[60]
17. Pancreatic juice[61,62]
18. Pericardial fluid[63,64]
19. Saliva[65-71]
20. Seminal fluid[72-76]
21. Sputum[77,78]
22. Sweat[79-81]
23. Synovial fluid[82-85]
24. Tears[86-91]
25. Urine[92-108]

*We apologize to all researchers whose excellent studies on these and other body fluids missed our attention, and were not included in this study.

1. Romero, R. et al. Proteomic analysis of amniotic fluid to identify women with preterm labor and intra-amniotic inflammation/infection: the use of a novel computational method to analyze mass spectrometric profiling. *J Matern Fetal Neonatal Med* **21**, 367-88 (2008).
2. Michaels, J.E. et al. Comprehensive proteomic analysis of the human amniotic fluid proteome: gestational age-dependent changes. *J Proteome Res* **6**, 1277-85 (2007).
3. Liu, X., Song, Y., Guo, Z., Sun, W. & Liu, J. A comprehensive profile and inter-individual variations analysis of the human normal amniotic fluid proteome. *J Proteomics* **192**, 1-9 (2019).
4. Li, X. et al. Peptidomic Analysis of Amniotic Fluid for Identification of Putative Bioactive Peptides in Ventricular Septal Defect. *Cell Physiol Biochem* **38**, 1999-2014 (2016).
5. Jeon, H.S. et al. Proteomic biomarkers in mid-trimester amniotic fluid associated with adverse pregnancy outcomes in patients with systemic lupus erythematosus. *PLoS One* **15**, e0235838 (2020).
6. Hallingstrom, M. et al. Mid-trimester amniotic fluid proteome's association with spontaneous preterm delivery and gestational duration. *PLoS One* **15**, e0232553 (2020).
7. Dixon, C.L. et al. Amniotic Fluid Exosome Proteomic Profile Exhibits Unique Pathways of Term and Preterm Labor. *Endocrinology* **159**, 2229-2240 (2018).
8. Cho, C.K., Shan, S.J., Winsor, E.J. & Diamandis, E.P. Proteomics analysis of human amniotic fluid. *Mol Cell Proteomics* **6**, 1406-15 (2007).
9. Bhatti, G. et al. The amniotic fluid proteome changes with gestational age in normal pregnancy: a cross-sectional study. *Sci Rep* **12**, 601 (2022).
10. Shender, V.O. et al. Proteome-metabolome profiling of ovarian cancer ascites reveals novel components involved in intercellular communication. *Mol Cell Proteomics* **13**, 3558-71 (2014).
11. Hoskins, E.R. et al. Proteomic analysis of ovarian cancer proximal fluids: validation of elevated peroxiredoxin 1 in patient peripheral circulation. *PLoS One* **6**, e25056 (2011).
12. Elschenbroich, S. et al. In-depth proteomics of ovarian cancer ascites: combining shotgun proteomics and selected reaction monitoring mass spectrometry. *J Proteome Res* **10**, 2286-99 (2011).
13. Guerrier, L. et al. Contribution of solid-phase hexapeptide ligand libraries to the repertoire of human bile proteins. *J Chromatogr A* **1176**, 192-205 (2007).
14. Farina, A. et al. Proteomic analysis of human bile from malignant biliary stenosis induced by pancreatic cancer. *J Proteome Res* **8**, 159-69 (2009).
15. Barbhuiya, M.A. et al. Comprehensive proteomic analysis of human bile. *Proteomics* **11**, 4443-53 (2011).
16. Farrah, T. et al. A high-confidence human plasma proteome reference set with estimated concentrations in PeptideAtlas. *Mol Cell Proteomics* **10**, M110 006353 (2011).
17. Geyer, P.E. et al. Plasma Proteome Profiling to Assess Human Health and Disease. *Cell Syst* **2**, 185-95 (2016).
18. Harel, M., Oren-Giladi, P., Kaidar-Person, O., Shaked, Y. & Geiger, T. Proteomics of microparticles with SILAC Quantification (PROMIS-Quan): a novel proteomic method for plasma biomarker quantification. *Mol Cell Proteomics* **14**, 1127-36 (2015).
19. Park, J. et al. In-depth blood proteome profiling analysis revealed distinct functional characteristics of plasma proteins between severe and non-severe COVID-19 patients. *Sci Rep* **10**, 22418 (2020).

20. Zeng, Z. et al. A proteomics platform combining depletion, multi-lectin affinity chromatography (M-LAC), and isoelectric focusing to study the breast cancer proteome. *Anal Chem* **83**, 4845-54 (2011).
21. Dey, K.K. et al. Deep undepleted human serum proteome profiling toward biomarker discovery for Alzheimer's disease. *Clin Proteomics* **16**, 16 (2019).
22. Liu, C.W. et al. Temporal expression profiling of plasma proteins reveals oxidative stress in early stages of Type 1 Diabetes progression. *J Proteomics* **172**, 100-110 (2018).
23. Garcia-Silva, S. et al. Use of extracellular vesicles from lymphatic drainage as surrogate markers of melanoma progression and BRAF (V600E) mutation. *J Exp Med* **216**, 1061-1070 (2019).
24. Sim, S.Y. et al. In-Depth Proteomic Analysis of Human Bronchoalveolar Lavage Fluid toward the Biomarker Discovery for Lung Cancers. *Proteomics Clin Appl* **13**, e1900028 (2019).
25. Shaba, E. et al. Proteome Characterization of BALF Extracellular Vesicles in Idiopathic Pulmonary Fibrosis: Unveiling Undercover Molecular Pathways. *Int J Mol Sci* **22**(2021).
26. Foster, M.W. et al. Quantitative proteomics of bronchoalveolar lavage fluid in idiopathic pulmonary fibrosis. *J Proteome Res* **14**, 1238-49 (2015).
27. Carvalho, A.S. et al. Bronchoalveolar Lavage Proteomics in Patients with Suspected Lung Cancer. *Sci Rep* **7**, 42190 (2017).
28. Zhang, J. et al. In-depth proteomic analysis of tissue interstitial fluid for hepatocellular carcinoma serum biomarker discovery. *Br J Cancer* **117**, 1676-1684 (2017).
29. Stone, M.D. et al. Novel In Situ Collection of Tumor Interstitial Fluid from a Head and Neck Squamous Carcinoma Reveals a Unique Proteome with Diagnostic Potential. *Clin Proteomics* **6**, 75-82 (2010).
30. Haslene-Hox, H. et al. Increased WD-repeat containing protein 1 in interstitial fluid from ovarian carcinomas shown by comparative proteomic analysis of malignant and healthy gynecological tissue. *Biochim Biophys Acta* **1834**, 2347-59 (2013).
31. Chiasserini, D. et al. Proteomic analysis of cerebrospinal fluid extracellular vesicles: a comprehensive dataset. *J Proteomics* **106**, 191-204 (2014).
32. Guha, D. et al. Proteomic analysis of cerebrospinal fluid extracellular vesicles reveals synaptic injury, inflammation, and stress response markers in HIV patients with cognitive impairment. *J Neuroinflammation* **16**, 254 (2019).
33. Macron, C., Lane, L., Nunez Galindo, A. & Dayon, L. Deep Dive on the Proteome of Human Cerebrospinal Fluid: A Valuable Data Resource for Biomarker Discovery and Missing Protein Identification. *J Proteome Res* **17**, 4113-4126 (2018).
34. Macron, C., Lane, L., Nunez Galindo, A. & Dayon, L. Identification of Missing Proteins in Normal Human Cerebrospinal Fluid. *J Proteome Res* **17**, 4315-4319 (2018).
35. Mouton-Barbosa, E. et al. In-depth exploration of cerebrospinal fluid by combining peptide ligand library treatment and label-free protein quantification. *Mol Cell Proteomics* **9**, 1006-21 (2010).
36. Muraoka, S. et al. Proteomic Profiling of Extracellular Vesicles Isolated From Cerebrospinal Fluid of Former National Football League Players at Risk for Chronic Traumatic Encephalopathy. *Front Neurosci* **13**, 1059 (2019).
37. Schutzer, S.E. et al. Establishing the proteome of normal human cerebrospinal fluid. *PLoS One* **5**, e10980 (2010).
38. Feig, M.A., Hammer, E., Volker, U. & Jehmlich, N. In-depth proteomic analysis of the human cerumen-a potential novel diagnostically relevant biofluid. *J Proteomics* **83**, 119-29 (2013).

39. Starodubtseva, N.L. et al. Label-free cervicovaginal fluid proteome profiling reflects the cervix neoplastic transformation. *J Mass Spectrom* **54**, 693-703 (2019).
40. Muytjens, C.M.J., Yu, Y. & Diamandis, E.P. Discovery of Antimicrobial Peptides in Cervical-Vaginal Fluid from Healthy Nonpregnant Women via an Integrated Proteome and Peptidome Analysis. *Proteomics* **17**(2017).
41. Kim, Y.E., Kim, K., Oh, H.B., Lee, S.K. & Kang, D. Quantitative proteomic profiling of Cervicovaginal fluid from pregnant women with term and preterm birth. *Proteome Sci* **19**, 3 (2021).
42. Muccilli, V. et al. Protein profile of exhaled breath condensate determined by high resolution mass spectrometry. *J Pharm Biomed Anal* **105**, 134-149 (2015).
43. Ma, L., Muscat, J.E., Sinha, R., Sun, D. & Xiu, G. Proteomics of exhaled breath condensate in lung cancer and controls using data-independent acquisition (DIA): a pilot study. *J Breath Res* **15**(2021).
44. Lacombe, M. et al. Proteomic characterization of human exhaled breath condensate. *J Breath Res* **12**, 021001 (2018).
45. Park, J.O. et al. Identification and characterization of proteins isolated from microvesicles derived from human lung cancer pleural effusions. *Proteomics* **13**, 2125-34 (2013).
46. Mundt, F. et al. Proteome screening of pleural effusions identifies galectin 1 as a diagnostic biomarker and highlights several prognostic biomarkers for malignant mesothelioma. *Mol Cell Proteomics* **13**, 701-15 (2014).
47. Liu, P.J. et al. In-depth proteomic analysis of six types of exudative pleural effusions for nonsmall cell lung cancer biomarker discovery. *Mol Cell Proteomics* **14**, 917-32 (2015).
48. Dzieciatkowska, M. et al. Proteomic analysis of human mesenteric lymph. *Shock* **35**, 331-8 (2011).
49. Dzieciatkowska, M. et al. Lymph is not a plasma ultrafiltrate: a proteomic analysis of injured patients. *Shock* **42**, 485-498 (2014).
50. Clement, C.C. et al. Protein expression profiles of human lymph and plasma mapped by 2D-DIGE and 1D SDS-PAGE coupled with nanoLC-ESI-MS/MS bottom-up proteomics. *J Proteomics* **78**, 172-87 (2013).
51. Zhang, Q. et al. Quantitative Analysis of the Human Milk Whey Proteome Reveals Developing Milk and Mammary-Gland Functions across the First Year of Lactation. *Proteomes* **1**, 128-158 (2013).
52. van Herwijnen, M.J. et al. Comprehensive Proteomic Analysis of Human Milk-derived Extracellular Vesicles Unveils a Novel Functional Proteome Distinct from Other Milk Components. *Mol Cell Proteomics* **15**, 3412-3423 (2016).
53. Molinari, C.E. et al. Proteome mapping of human skim milk proteins in term and preterm milk. *J Proteome Res* **11**, 1696-714 (2012).
54. Goonatilleke, E. et al. Human Milk Proteins and Their Glycosylation Exhibit Quantitative Dynamic Variations during Lactation. *J Nutr* **149**, 1317-1325 (2019).
55. Dayon, L., Macron, C., Lahrichi, S., Nunez Galindo, A. & Affolter, M. Proteomics of Human Milk: Definition of a Discovery Workflow for Clinical Research Studies. *J Proteome Res* **20**, 2283-2290 (2021).
56. Beck, K.L. et al. Comparative Proteomics of Human and Macaque Milk Reveals Species-Specific Nutrition during Postnatal Development. *J Proteome Res* **14**, 2143-57 (2015).
57. Pavlou, M.P., Kulasingam, V., Sauter, E.R., Kliethermes, B. & Diamandis, E.P. Nipple aspirate fluid proteome of healthy females and patients with breast cancer. *Clin Chem* **56**, 848-55 (2010).

58. Mannello, F. New horizon for breast cancer biomarker discoveries: What might the liquid biopsy of nipple aspirate fluid hold? *Proteomics Clin Appl* **11**(2017).
59. Brunoro, G.V.F. et al. Differential proteomic comparison of breast cancer secretome using a quantitative paired analysis workflow. *BMC Cancer* **19**, 365 (2019).
60. Cuoghi, A. et al. Role of proteomics to differentiate between benign and potentially malignant pancreatic cysts. *J Proteome Res* **10**, 2664-70 (2011).
61. Paulo, J.A. et al. Analysis of endoscopic pancreatic function test (ePFT)-collected pancreatic fluid proteins precipitated via ultracentrifugation. *JOP* **14**, 176-86 (2013).
62. Marchegiani, G., Paulo, J.A., Sahora, K. & Fernandez-Del Castillo, C. The proteome of postsurgical pancreatic juice. *Pancreas* **44**, 574-82 (2015).
63. Xiang, F. et al. Proteomics analysis of human pericardial fluid. *Proteomics* **13**, 2692-5 (2013).
64. Ullah, J., Hashmi, S., Ali, A., Khan, F., Sami, S.A., Bokhari, S.S., Sharif, H., El-Seedi, H.R. Musharraf, S.G. Pericardial fluid proteomic label-free quantification of differentially expressed proteins in ischemic heart disease patients with systolic dysfunction by nano-LC-ESI-MS/MS analysis. *RSC Adv.* **11**, 320-327 (2021).
65. Bandhakavi, S., Stone, M.D., Onsongo, G., Van Riper, S.K. & Griffin, T.J. A dynamic range compression and three-dimensional peptide fractionation analysis platform expands proteome coverage and the diagnostic potential of whole saliva. *J Proteome Res* **8**, 5590-600 (2009).
66. Fang, X. et al. Comparison of electrokinetics-based multidimensional separations coupled with electrospray ionization-tandem mass spectrometry for characterization of human salivary proteins. *Anal Chem* **79**, 5785-92 (2007).
67. Sivadasan, P. et al. Human salivary proteome--a resource of potential biomarkers for oral cancer. *J Proteomics* **127**, 89-95 (2015).
68. Sivadasan, P. et al. Data from human salivary proteome - A resource of potential biomarkers for oral cancer. *Data Brief* **4**, 374-8 (2015).
69. Sun, Y. et al. Comparative Proteomic Analysis of Exosomes and Microvesicles in Human Saliva for Lung Cancer. *J Proteome Res* **17**, 1101-1107 (2018).
70. Wu, C.C., Chu, H.W., Hsu, C.W., Chang, K.P. & Liu, H.P. Saliva proteome profiling reveals potential salivary biomarkers for detection of oral cavity squamous cell carcinoma. *Proteomics* **15**, 3394-404 (2015).
71. Yan, W. et al. Systematic comparison of the human saliva and plasma proteomes. *Proteomics Clin Appl* **3**, 116-134 (2009).
72. Agarwal, A. et al. Comparative proteomic network signatures in seminal plasma of infertile men as a function of reactive oxygen species. *Clin Proteomics* **12**, 23 (2015).
73. Ronquist, K.G. et al. Prostasomes from four different species are able to produce extracellular adenosine triphosphate (ATP). *Biochim Biophys Acta* **1830**, 4604-10 (2013).
74. Yang, C. et al. Comprehensive proteomics analysis of exosomes derived from human seminal plasma. *Andrology* **5**, 1007-1015 (2017).
75. Zhang, X., Vos, H.R., Tao, W. & Stoorvogel, W. Proteomic Profiling of Two Distinct Populations of Extracellular Vesicles Isolated from Human Seminal Plasma. *Int J Mol Sci* **21**(2020).
76. Pilch, B. & Mann, M. Large-scale and high-confidence proteomic analysis of human seminal plasma. *Genome Biol* **7**, R40 (2006).
77. HaileMariam, M. et al. Protein and Microbial Biomarkers in Sputum Discern Acute and Latent Tuberculosis in Investigation of Pastoral Ethiopian Cohort. *Front Cell Infect Microbiol* **11**, 595554 (2021).

78. Burg, D. et al. Large-Scale Label-Free Quantitative Mapping of the Sputum Proteome. *J Proteome Res* **17**, 2072-2091 (2018).
79. Yu, Y., Prassas, I., Muytjens, C.M. & Diamandis, E.P. Proteomic and peptidomic analysis of human sweat with emphasis on proteolysis. *J Proteomics* **155**, 40-48 (2017).
80. Raiszadeh, M.M. et al. Proteomic analysis of eccrine sweat: implications for the discovery of schizophrenia biomarker proteins. *J Proteome Res* **11**, 2127-39 (2012).
81. Burian, M. et al. Quantitative proteomics of the human skin secretome reveal a reduction in immune defense mediators in ectodermal dysplasia patients. *J Invest Dermatol* **135**, 759-767 (2015).
82. Foers, A.D. et al. Proteomic analysis of extracellular vesicles reveals an immunogenic cargo in rheumatoid arthritis synovial fluid. *Clin Transl Immunology* **9**, e1185 (2020).
83. Bhattacharjee, M. et al. Synovial fluid proteome in rheumatoid arthritis. *Clin Proteomics* **13**, 12 (2016).
84. Balakrishnan, L. et al. Proteomic analysis of human osteoarthritis synovial fluid. *Clin Proteomics* **11**, 6 (2014).
85. Anderson, J.R. et al. The synovial fluid proteome differentiates between septic and nonseptic articular pathologies. *J Proteomics* **202**, 103370 (2019).
86. Zhou, L. et al. In-depth analysis of the human tear proteome. *J Proteomics* **75**, 3877-85 (2012).
87. Tong, L. et al. Quantitation of 47 human tear proteins using high resolution multiple reaction monitoring (HR-MRM) based-mass spectrometry. *J Proteomics* **115**, 36-48 (2015).
88. Nattinen, J. et al. Early changes in tear film protein profiles after femtosecond LASIK surgery. *Clin Proteomics* **17**, 36 (2020).
89. Dor, M. et al. Investigation of the global protein content from healthy human tears. *Exp Eye Res* **179**, 64-74 (2019).
90. Cheung, J.K. et al. Human tear proteome dataset in response to daily wear of water gradient contact lens using SWATH-MS approach. *Data Brief* **36**, 107120 (2021).
91. Chen, X. et al. Integrated Tear Proteome and Metabolome Reveal Panels of Inflammatory-Related Molecules via Key Regulatory Pathways in Dry Eye Syndrome. *J Proteome Res* **18**, 2321-2330 (2019).
92. Adachi, J., Kumar, C., Zhang, Y., Olsen, J.V. & Mann, M. The human urinary proteome contains more than 1500 proteins, including a large proportion of membrane proteins. *Genome Biol* **7**, R80 (2006).
93. Li, Q.R. et al. A comprehensive and non-prefractionation on the protein level approach for the human urinary proteome: touching phosphorylation in urine. *Rapid Commun Mass Spectrom* **24**, 823-32 (2010).
94. Marimuthu, A. et al. A comprehensive map of the human urinary proteome. *J Proteome Res* **10**, 2734-43 (2011).
95. Santucci, L. et al. From hundreds to thousands: Widening the normal human Urinome (1). *J Proteomics* **112**, 53-62 (2015).
96. Zhao, M. et al. A comprehensive analysis and annotation of human normal urinary proteome. *Sci Rep* **7**, 3024 (2017).
97. Zheng, J., Liu, L., Wang, J. & Jin, Q. Urinary proteomic and non-prefractionation quantitative phosphoproteomic analysis during pregnancy and non-pregnancy. *BMC Genomics* **14**, 777 (2013).
98. Gonzales, P.A. et al. Large-scale proteomics and phosphoproteomics of urinary exosomes. *J Am Soc Nephrol* **20**, 363-79 (2009).

99. Hogan, M.C. et al. Subfractionation, characterization, and in-depth proteomic analysis of glomerular membrane vesicles in human urine. *Kidney Int* **85**, 1225-37 (2014).
100. Lin, L., Yu, Q., Zheng, J., Cai, Z. & Tian, R. Fast quantitative urinary proteomic profiling workflow for biomarker discovery in kidney cancer. *Clin Proteomics* **15**, 42 (2018).
101. Nielsen, H.H. et al. The Urine Proteome Profile Is Different in Neuromyelitis Optica Compared to Multiple Sclerosis: A Clinical Proteome Study. *PLoS One* **10**, e0139659 (2015).
102. Prikryl, P. et al. Mass spectrometry-based proteomic exploration of the small urinary extracellular vesicles in ANCA-associated vasculitis in comparison with total urine. *J Proteomics* **233**, 104067 (2021).
103. Principe, S. et al. Identification of prostate-enriched proteins by in-depth proteomic analyses of expressed prostatic secretions in urine. *J Proteome Res* **11**, 2386-96 (2012).
104. Wang, Z., Hill, S., Luther, J.M., Hachey, D.L. & Schey, K.L. Proteomic analysis of urine exosomes by multidimensional protein identification technology (MudPIT). *Proteomics* **12**, 329-38 (2012).
105. Huo, S. et al. Urinary Proteomic Characteristics of Hyperuricemia and Their Possible Links with the Occurrence of Its Concomitant Diseases. *ACS Omega* **6**, 9500-9508 (2021).
106. Li, Y. et al. Urine proteome of COVID-19 patients. *Urine (Amst)* **2**, 1-8 (2020).
107. Swensen, A.C. et al. A Comprehensive Urine Proteome Database Generated From Patients With Various Renal Conditions and Prostate Cancer. *Front Med (Lausanne)* **8**, 548212 (2021).
108. Wang, S., Kojima, K., Mobley, J.A. & West, A.B. Proteomic analysis of urinary extracellular vesicles reveal biomarkers for neurologic disease. *EBioMedicine* **45**, 351-361 (2019).
